# Surgery Under General Anesthesia Alleviated the Hyperactivity but Had No Effect on the Susceptibility to PND in ADHD Rats

**DOI:** 10.3389/fpsyt.2019.00642

**Published:** 2019-09-03

**Authors:** Peng Zhang, Feifei Xu, Guangchao Zhao, Xinxin Zhang, Ao Li, Hailong Dong, Lize Xiong

**Affiliations:** Department of Anesthesiology and Perioperative Medicine, Xijing Hospital, the Fourth Military Medical University, Xi’an, China

**Keywords:** ADHD, postoperative neurocognitive disorder, surgery, general anesthesia, nucleus accumbens

## Abstract

**Background:** Attention-deficit hyperactivity disorder (ADHD) is a typical neuropsychiatric disorder characterized by inattention, impulsivity, and hyperactivity, particularly in children. Recent studies demonstrated a close relationship between the development of ADHD and surgery under general anesthesia. However, few studies illustrated if ADHD symptoms changed after surgery. Meanwhile, whether these individuals with natural neural impairment were sensitive to postoperative neurocognitive disorder (PND) still remain unclear.

**Methods:** Spontaneously hypertensive rats (SHR) were utilized as spontaneous ADHD animal model and Wistar-Kyoto (WKY) rats as non-ADHD animal model. We evaluated the variation of neurocognitive function and locomotor activity of the rats undergoing experimental laparotomy with general anesthesia by isoflurane. Neurocognitive function was assessed by fear conditioning test for contextual memory and Morris water maze (MWM) for spatial memory. Depressive-like behavior after surgery was detected by forced swim test, and open-field test and elevated plus maze test were utilized to evaluate locomotor activities and anxiety. Furthermore, we compared electroencephalogram (EEG) signal in ADHD and WKY rats under free-moving conditions. Afterward, *c-Fos* staining was also utilized to detect the excitatory activity of neurons in these rats to explore the neural mechanism.

**Results:** Locomotor activity of SHR assessed by average speed and number of line crossings in the open-field test decreased 1 week after surgery under general anesthesia, but there was no difference concerning anxiety levels between SHR and WKY rats after surgery. This phenomenon was also paralleled with the change in EEG signal (delta band 0∼3 Hz). Surgery under general anesthesia had no effect on spatial and contextual memory, while it improved spontaneous depression in SHR. The expression of *c-Fos* was downregulated for at least 1 week in the nucleus accumbens (NAc) area of ADHD rats’ brain after surgery.

**Conclusion:** ADHD rats were not sensitive to PND. Surgery with general anesthesia could partly improve the hyperactivity symptom of ADHD rats. This mechanism was related to the suppression of neural activity in the cerebral NAc of ADHD rats induced by general anesthetics.

## Introduction

Attention-deficit hyperactivity disorder (ADHD) is a typical and heterogeneous neuropsychiatric disorder characterized by impaired levels of hyperactivity, impulsivity, and inattention (1), which affects children in particular. In earlier studies, ADHD had been proved as a central nervous system (CNS) disorder induced by gene polymorphism and imbalance of excitatory and inhibitory neurotransmitters (2). Psychiatric Genomics Consortium genome-wide association study (GWAS), including 20,183 individuals with ADHD and 35,191 controls, provides large-scale data on how common genetic variations are associated with the ADHD (3). The etiology of ADHD was, however, not fully understood (4).

In 2016, the US Food and Drug Administration issued a warning regarding impaired brain development in children following exposure to certain anesthetic agents used for general anesthesia, namely, the inhalational anesthetics isoflurane, sevoflurane, and desflurane and the intravenous agents propofol and midazolam, in the third trimester of pregnancy (5). It indicated that there would be a close interaction between the development of this congenital neural disease and surgery under general anesthesia. However, few studies had illustrated if the symptom of ADHD changed following surgery (6). Meanwhile, whether these individuals with natural neural impairment were also sensitive to perioperative neural disorder still remained unclear. For instance, postoperative neurocognitive disorder (PND) was a severe complication that influences neural function, including compromised attention, memory, orientation, executive function, or language fluency after surgery (7). Hence, it is necessary to verify the impacts and safety of surgery and general anesthesia on ADHD patients.

In the current study, we utilized spontaneously hypertensive rats (SHR) as a natural ADHD model (8). Locomotor activity and neurocognitive behavioral performance were observed and electroencephalogram (EEG) signal under free-moving condition was monitored from preoperation to 1 week postoperation. We further compared the specific change in different nuclei among SHR and Wistar-Kyoto (WKY) rats after surgery. Such a research will help shed light on a new perspective for what the relations are between the surgery and ADHD.

## Materials and Methods

### Animals

We used the SHR as spontaneous ADHD animal model and the Wistar-Kyoto Rats (WKY) as non-ADHD animal model. All animal procedures were carried out in accordance with the National Institutes of Health Guide for the Care and Use of Laboratory Animals, and experimental protocols were approved by the Ethics Committee for Animal Experimentation of the Fourth Military Medical University. Animals were habituated for 1 week before any intervention with access to water and food *ad libitum* and kept on a standard 12-h light/12-h dark cycle.

### Experimental Design

All the rats were bought at the age of 6 weeks. After 1 week habituation, we divided both the SHR and WKY rats into surgery group and nonsurgery group and implemented the animal surgery and corresponding control procedure. Then, at the time point of 1 week postoperation, we evaluated the locomotive activity, contextual memory, spatial memory, EEG, and *c-Fos* expression of all rats. Except for EEG signal monitoring, rats in no-surgery group and surgery group were independently enrolled and treated nonconsecutively. We utilized different batches of rats at every individual experiment.

### Experimental Laparotomy

Experimental laparotomy was performed on animals with general anesthesia by isoflurane to evaluate the variation of neurocognitive function and locomotor activity after surgery. Anesthesia was performed through a face mask (1.5 to 2.0% isoflurane, O2 1.0 L/min). Animals were placed on a heating pad during the surgery to keep the body temperature between 36.5 and 37.0°C. The abdominal hair was shaved, and the skin was sterilized. A 2-cm incision was performed on the midline of the abdomen. Approximately 5-cm small intestine was exteriorized from the peritoneal cavity, covered with gauze soaked with normal saline, and gently rubbed for 10 min. After the manipulation, abdominal muscle was closed continuously with 5-0 Vicryl sutures (Polysorb^TM^, U.S.A.), followed by skin interrupted closure with 4-0 silk suture. Ropivacaine/lidocaine 0.2% (300 μl) was locally injected for postoperative analgesia to avoid the impact of pain to neurocognitive assessment. The surgery duration was controlled at approximately 30 min. Postoperative animals would recover in an incubator at 35°C for 30 min, then return to their home cages.

### Neurocognitive Function Assessment

Behavioral tests were applied to assess locomotor activity, depression, anxiety, contextual memory, and spatial memory according to previous protocols with slight modifications (9–12).

#### Fear Conditioning Test

The fear conditioning test consists of three phases: habituation phase, training phase, and test phase. On training day, five times of foot shocks were delivered (current: 0.7 mA, 2 s; interval between each foot shock: 35–60 s). Twenty-four hours later, rats were kept in the same context for 5 min for assessment of contextual memory retrieval. The animals were considered freezing if no movement was detected for 2 s.

#### Morris Water Maze

The Morris water maze (MWM) was carried out as described before with modifications. The circular water pool (150 cm diameter and 80 cm high) was placed in a soundproof test room. The pool was filled with water (22 ± 1°C) to a height of 42 cm, and the pool was artificially divided into four conceptual quadrants (N, S, E, or W). Each quadrant was designed as a starting point in the subsequent sessions. A platform (20 cm diameter and 40 cm high) was set inside and fixed in the middle of S quadrant, submerged 2 cm below the water surface. In each session, animals were placed in the water at one of the four starting points. Rats underwent four trials per day at 50-min intervals, repeated for 5 training days. Each rat was given 90 s to search for the platform. If the rat failed to do so independently, it would be guided to the platform and left to stay there for 30 s before returning to its home cage. In the memory trials, if the rat could not find the platform in 90 s, the session was finished and the maximum score of 90 s was recorded. Learning and memory trials were recorded using an overhead video camera (Sony DCR-SR85) at the center of the pool. The variables measured were the time to get the platform (or the time taken to cross the site of the platform) and the time spent on the platform quadrant (or spent in the quadrant where the platform was placed during training sessions).

#### Open-Field Test

Animals were placed in an open-field apparatus (120 cm long *120 cm wide *40 cm high) for free exploring the field for 5 min. All of the moving traces were recorded by an overhead camera. The total travel distance was analyzed by Video Tracking Software (ANY-maze, Stoelting Co., Ltd.). At the end of each session, the surface and side walls of the apparatus were cleaned with ethanol before and after each session to eliminate any olfactory cues that may affect the outcomes of subsequent sessions.

#### Elevated Plus Maze Test

The elevated plus maze apparatus consists of a central platform (10 cm long * 10 cm wide), two open arms (50 cm long * 10 cm wide), and two closed arms with protective walls of 40 cm high (50 cm long * 10 cm wide) that is 50 cm above the ground. Animals were placed in the central platform facing one open arm of the apparatus and were free to explore the arms for 5 min. The apparatus was cleaned with ethanol before and after each session. All of the traces were recorded by an overhead camera. The travel time and numbers of entry into the open/closed arms were analyzed by Video Tracking Software (ANY-maze, Stoelting Co., Ltd.).

### Forced Swim Test

Animals were placed in a 60 × 20 cm Plexiglas cylinder containing 30 cm of water (maintained at 23–25°C) and forced to swim for 15 min. Animals were re-placed in the water on the next day for 5 min, and all of the behaviors were recorded by a front camera. The immobile time (floating and necessary movements to breathe) was assessed by Video Tracking Software (ANY-maze, Stoelting Co., Ltd.).

### EEG Signal Monitoring

Electrodes were implanted on the scalps of animals at 5 weeks of age, and then they were allowed to recover for 5–7 days after electrode implantation. After rehabilitation for 1 week, EEG recording was performed at 6–7 weeks of age. Three EEG electrodes were implanted on the left and right frontal (± 2.0 mm lateral and 3.2 mm anterior from the bregma), parietal (± 3.5 mm lateral and 1.8 mm posterior from the bregma), and occipital cortex (± 2.0 mm lateral and 5.2 mm posterior from the bregma) of the scalps. The left occipital cortex electrode was used as a reference. One week after experimental laparotomy, rats in the same group were rehabilitation for another 1 week and then monitored EEG signal. With this setup, five times series of EEG recordings were obtained.

### Immunohistochemistry

Animals were deeply anesthetized with overdose chloral hydrate (80 mg/kg, i.p.), cardiac perfused with ice-cold phosphate-buffered saline (PBS), and followed by 4% formaldehyde solution. The brains were harvested from the skull and then postfixed for at least 24 h. After postfixation, each brain was sliced coronally (30 mm thickness) using a microslicer (DSK-3000, Dosaka, Kyoto, Japan). The *Fos*-IR staining was performed using a previous protocol with adjustments (Ohno et al., 2009a, 2011). Briefly, brain slices were washed in PBS with 0.3% Triton X-100, incubated for 2 h in 2% normal rabbit serum, and then incubated again in the presence of goat c-Fos antibody and 2% normal rabbit serum (Santa Cruz Biotechnology Inc., Santa Cruz, CA) for 18–36 h. The sections were then washed in PBS and incubated with the biotinylated rabbit anti-goat IgG secondary antibody (Vector Laboratories, Burlingame, CA) for another 2 h. After incubating with the secondary antibody, brain sections were then incubated with PBS containing 0.3% hydrogen peroxide for 30 min. At last, the sections were cleaned with PBS and incubated for 2 h using an avidin-biotinylated horseradish peroxidase complex (Vectastain ABC Kit). The diaminobenzidine-nickel staining method was performed for visualization of *Fos* staining.

### Statistical Analysis

Statistical analysis was performed using the SPSS version 20.0 program (SPSS Inc., Chicago, IL) or GraphPad Prism 7.0 software. For comparison of locomotor activity and neurocognitive tests between two groups, Student’s t-test was used. A one-way analysis of variance (ANOVA) with *post hoc* Tukey’s test was used when more than two groups were compared. Freezing time ratio was analyzed with Mann-Whitney U test when comparing two groups, whereas Kruskal-Wallis test with Dunn’s *post hoc* test was used when comparing four groups. For spatial learning (escape latency), a two-way ANOVA was used to determine statistical significance among groups at different time points with Sidak’s multiple comparison. P < 0.05 was considered significant.

## Results

### Experimental Laparotomy Reduced Locomotor Activity in SHR and Had No Effect on Anxiety

**Figure 1:**
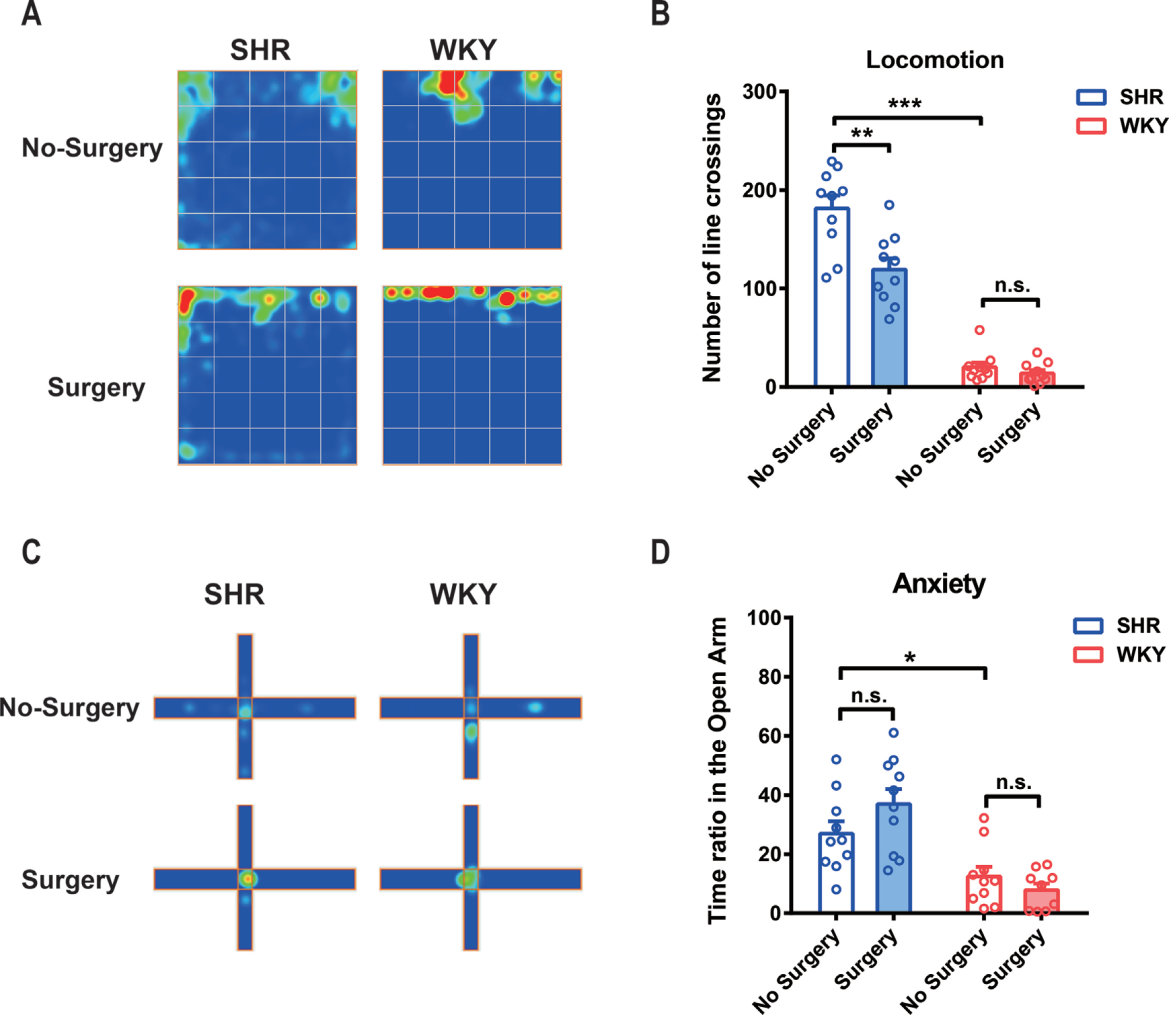
Experimental laparotomy reduced locomotor activity in SHR rats and had no effect on anxiety. **(A)** Average heatmap of open-field test. **(B)** Statistical histogram of locomotor activity in the open field. Number of line crossings was calculated for assessing locomotor activities. SHR-No Surgery vs. WKY-No Surgery, ***P < 0.0001; SHR-No Surgery vs. SHR-Surgery, **P = 0.0002; WKY-No Surgery vs. WKY-Surgery, *P* = 0.8502, Two-way ANOVA, n = 10. **(C)** Average heatmap of elevated plus maze test. **(D)** Statistical histogram of anxiety situation in elevated plus maze. Time ratios in the open arm were calculated for assessing anxiety situation. SHR-No surgery vs. WKY-No Surgery, *P = 0.0237; SHR-No Surgery vs. SHR-Surgery, *P* = 0.1398; WKY-No Surgery vs. WKY-Surgery, *P* = 0.6554, Two-way ANOVA, *n* = 10.

We used both SHR and WKY rats to investigate the variation of locomotor activity and emotion after surgery, especially in ADHD individuals. Locomotor activities, assessed by number of line crossings in open-field test, were significantly higher in SHR than that in WKY rats (**Figures 1A, B**; ***P < 0.0001, SHR-No Surgery vs. WKY-No Surgery). Little has changed in WKY rats after surgery. However, locomotor activities in SHR were reduced 1 week after surgery, and this reduction could last up to 2 weeks postoperation (**Figures 1A, B**; **P = 0.0002, No Surgery vs. Surgery in SHR group, *P* = 0.8502, No Surgery vs. Surgery in WKY group). Compared with SHR, WKY rats spent less time in the open arm of the elevated plus maze, which indicated that WKY rats were more apt to be anxious in physiology (**Figures 1C, D**; *P = 0.0237, SHR-No Surgery vs. WKY-No Surgery, 26.90 ± 5.44 vs. 12.47 ± 5.56). So, we further detected anxiety by utilizing elevated plus maze test again 1 week postoperation. Results showed no alteration in the time ratio in the open arm between Surgery and No-Surgery group in both SHR and WKY rats (**Figures 1C, D**; *P* > 0.05, No-Surgery vs. Surgery). Therefore, 1 week after surgery, the hyperactivity symptoms but not the anxiety of SHR were alleviated.

### Experimental Laparotomy Did Not Impair the Spatial and Contextual Memory, Whereas It Alleviated Spontaneous Depression in SHR

**Figure 2:**
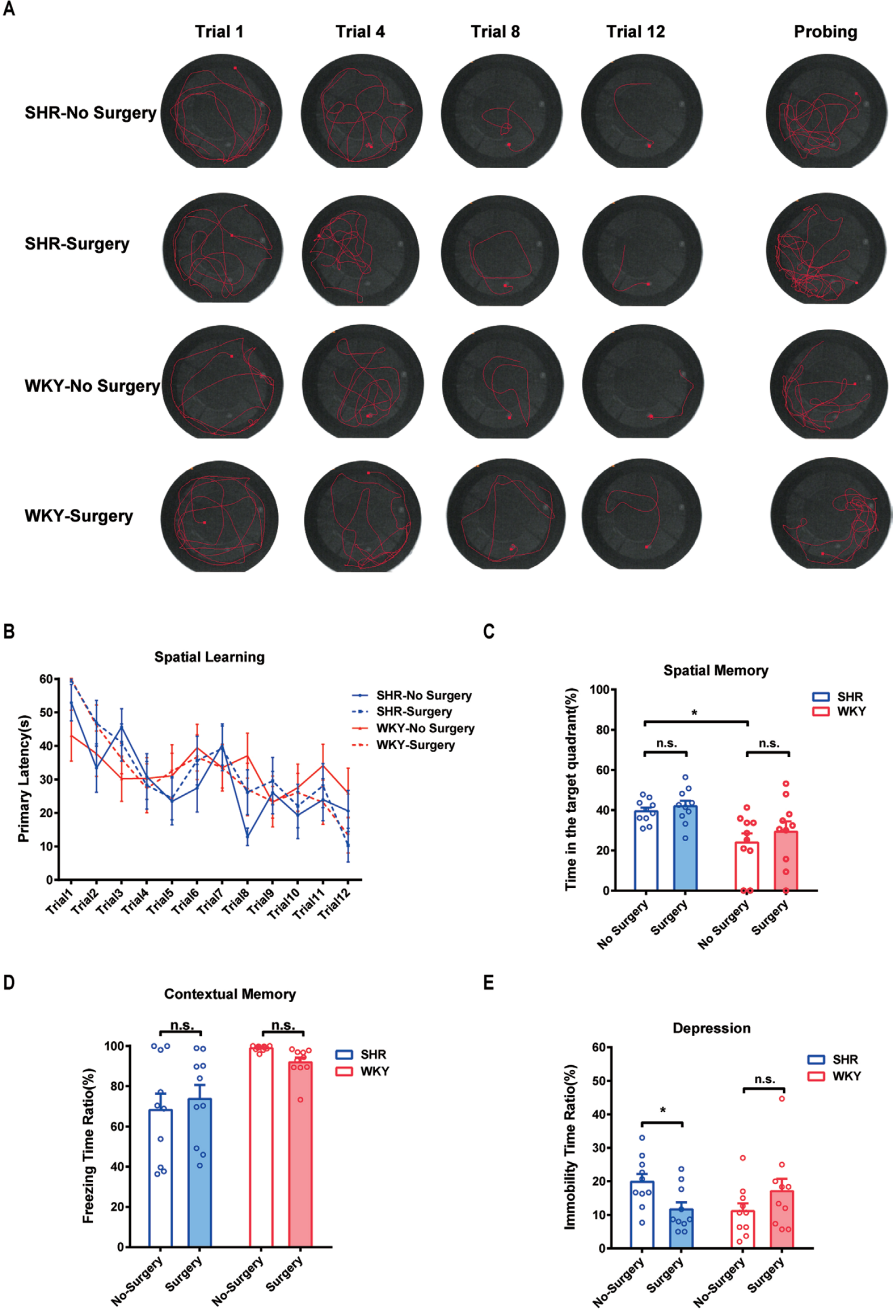
Experimental laparotomy did not impair the spatial and contextual memory but alleviated spontaneous depression in SHR. **(A)** Typical track maps of MWM training and testing. **(B)** Statistical line graph of spatial learning speed in MWM. Primary latencies to the escape platform were calculated for assessing spatial learning speed, Two-way ANOVA, *P* = 0.8820, SHR-No Surgery vs. SHR-Surgery. *P* = 0.9981, WKY-No Surgery vs. WKY-Surgery. **(C)** Statistical histogram of spatial memory in MWM test. Time ratios in the target quadrant (%) were calculated for assessing spatial memory. *P = 0.0140, SHR-No Surgery vs. WKY-No Surgery. *P* = 0.8735, SHR-No Surgery vs. SHR-Surgery. *P* = 0.5594, WKY-No Surgery vs. WKY-Surgery. **(D)** Statistical histogram of contextual memory in the fear conditioning test. Freezing time ratios (%) were calculated for assessing contextual memory. Two-way ANOVA, *P* = 0.7314, SHR-No Surgery vs. SHR-Surgery. *P* = 0.6037, WKY-No Surgery vs. WKY-Surgery. **(E)** Statistical histogram of depression in the forced swim test. Immobile time ratios (%) were calculated for assessing depressive situations. Two-way ANOVA, *P = 0.0172, SHR-No Surgery vs. SHR-Surgery. *N* = 10 in each group.

To testify whether ADHD individuals were more sensitive to PNDs, we utilized MWM test, fear conditioning test, and forced swim test to assess the spatial memory, contextual memory, and depression, respectively, in SHR and WKY rats. Our results showed experimental laparotomy did not cause any change in spatial memory in both SHR and WKY rats (**Figure 2A**, typical tracks of SHR and WKY rats in Morris Water Maze test. **Figure 2B**, No Surgery vs. Surgery in SHR, *P* = 0.8820; No Surgery vs. Surgery in WKY, *P* = 0.9981, *n* = 10 Two-way ANOVA, Tukey’s multiple comparisons test. **Figure 2C**, No Surgery vs. Surgery in SHR, *P* = 0.8735; No Surgery vs. Surgery in WKY, *P* = 0.5594, *n* = 10, Two-way ANOVA), although SHR had a better performance in the retrieval of spatial memory on probing day under the condition without surgery (**Figure 2C**, SHR-No Surgery vs. WKY-No Surgery, *P* = 0.0140, *n* = 10, Two-way ANOVA). Meanwhile, surgery did not affect the contextual memory of SHR nor did it affect WKY rats (**Figure 2D**, No Surgery vs. Surgery in SHR, *P* = 0.7314; No Surgery vs. Surgery in WKY, *P* = 0.6037, *n* = 10 Two-way ANOVA). Subsequently, we observed that surgery could reduce the immobility time of SHR in the forced swim test, suggesting that surgery with general anesthesia could improve depression of SHR (**Figure 2E**; *P = 0.0172, No Surgery vs. Surgery in SHR). These results indicated that SHR were not susceptible to PND whereas surgery with general anesthesia was safe for ADHD patients.

### Delta Band of EEG Signal Was Significantly Enhanced 1 Week After Surgery in SHR

**Figure 3:**
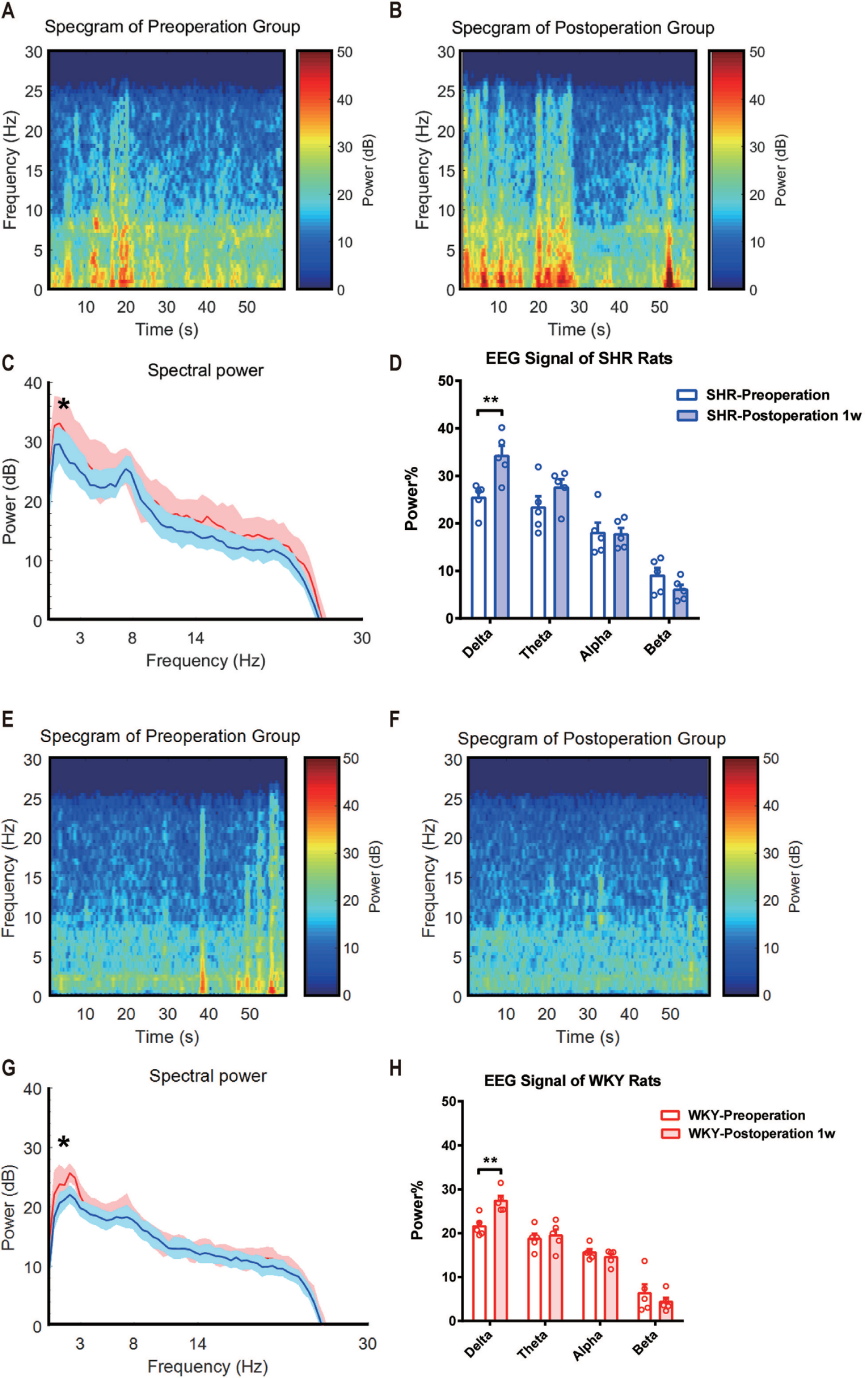
Delta band power of EEG signal was significantly enhanced after surgery in both SHR and WHR rats. **(A**, **E)** Average EEG spectrum of SHR and WKY rats before surgery. **(B**, **F)** Average EEG spectrum of SHR and WKY rats at 1 week after surgery. **(C**,**G)** Statistical line graph of EEG signals power. *P = 0.0124, SHR preoperation vs. SHR postoperation 1 week; *P = 0.0296, WKY preoperation vs. WKY postoperation 1 week. **(D**, **H)** Statistical histogram of EEG signals power in different bands. **P = 0.0062, SHR preoperation vs. SHR postoperation 1 week in delta band; **P = 0.0076, WKY preoperation vs. WKY postoperation 1 week in delta band. Delta band (0∼4 Hz). Two-way ANOVA, *N* = 5 in each group.

In earlier studies, it has been demonstrated that ADHD individuals performed an abnormal EEG signal (2, 13). Here, we monitored EEG signals of SHR and WKY rats under a free-moving condition before and 1 week after experimental laparotomy (**Figures 3A, B, E, F**). Compared with the preoperative signal, the power of EEG signal in Delta (0∼3 Hz) band significantly enhanced 1 week after surgery (**Figure 3C, G**. Statistical line graph of EEG signals power. *P = 0.0124, SHR preoperation vs. SHR postoperation 1 week; *P = 0.0296, WKY preoperation vs. WKY postoperation 1 week.) and the band ratio of EEG in delta band also increased (**Figure 3D, H**. Statistical histogram of EEG signals power in different bands. **P = 0.0062, SHR preoperation vs. SHR postoperation 1 week in delta band; **P = 0.0076, WKY preoperation vs. WKY postoperation 1 week in delta band). No variation observed in Theta (3∼8 Hz) band, Alpha band (8∼14 Hz), and Beta band (14∼30 Hz). These results suggested that the improvement of ADHD symptoms as hyperactivity induced by surgery was associated with the change in neural excitation.

### The C-Fos Expression of SHR Significantly Decreased in Nucleus Accumbens After Surgery

**Figure 4:**
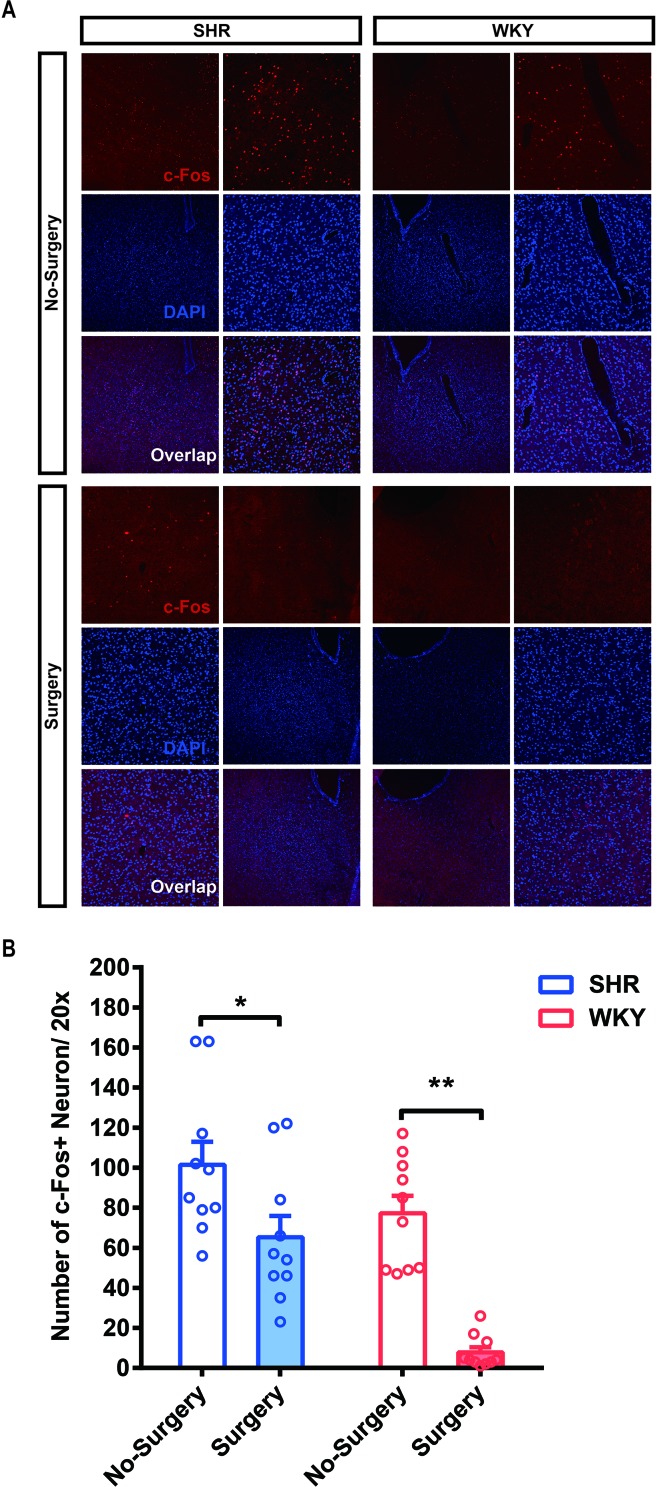
The *c-Fos* expression of SHR and WKY rats was significantly alleviated in the NAc (nucleus accumbens) 1 week after surgery. (A) *c-Fos* staining in the NAc area of SHR and WKY rats brain tissue between No-Surgery group and Surgery group. (B) Statistical histogram of neuronal excitation. Number of *c-Fos*+ neuron per 20× vision was calculated for assessing neuronal excitatory.*P < 0.05, SHR No-Surgery vs. SHR Surgery; **P < 0.01, WKY No-Surgery vs. WKY Surgery. Two-way ANOVA, N = 10 in each group.

To determine whether surgery under general anesthesia had a lasting impact on the function of neural circuits, we compared the neuronal excitability in nucleus accumbens (NAc)-related circuitry *via c-Fos* staining in both SHR and WKY rats. Before surgery, the expression of *c-*Fos in SHR was slightly higher than in WKY rats, but there was no statistical difference. After surgery, the expression of *c-Fos* in NAc region was significantly decreased, which would last for at least 1 week (**Figures 4A, B**; *P < 0.05, No Surgery vs. Surgery in SHR; **P < 0.05, No Surgery vs. Surgery in WKY rats). NAc nucleus was an important brain region associated with ADHD symptoms (8). The variation of neural activities in the NAc region induced by surgery was related to the behavioral improvement in ADHD individuals.

## Discussion

ADHD is one of the most common mental disorders affecting children, adolescents, and adults (14). In recent years, benefited from the combination of basic research and multicenter clinical research, the pathological mechanism and clinical therapeutics of ADHD gained great breakthroughs. In terms of a congenital nervous system disease, ADHD individuals not only suffered from typical symptoms, hyperactivity–impulsivity and inattention but also were sensitive to other neurocognitive disorders (15, 16). In the current study, we aimed to elucidate whether surgery under general anesthesia was safe for these ADHD individuals. Hence, we observed the locomotor activity, anxiety, neurocognitive behaviors, depression, activity of specific neural circuits and monitored EEG signal in ambulatory state from preoperation to 1 week postoperation. According to the results, we demonstrated that laparotomy under general anesthesia was safe for spontaneous ADHD rodents, SHR. More importantly, the locomotor hyperactivity even partly improved in ADHD individuals 1 week after surgery, and there was no evidence that SHR were susceptible to perioperative neurocognitive disorders.

Scientists proceeded with researches about the etiology of ADHD from the aspect of genomics, epigenetics, neurology, psychology, and so on. In our previous study, we demonstrated that deficiency of tumor suppressor NDRG2 could lead to attention deficit and hyperactive behavior in children by inhibition of astroglial glutamate clearance (2). On the other hand, in children who were diagnosed as having ADHD, the functions of DA and NE transporters were overly or lowly activated, which was considered to cause symptoms of attention deficit, impulsiveness, and hyperactivity (17, 18). Such findings were a compelling reminder that the imbalance of excitatory and inhibitory neurotransmitter homeostasis in the neurotransmitter system was a crucial influential factor for ADHD development. Thus, we hypothesized that the imbalance of the neurotransmitter system caused by any stimulus was at risk of aggravating ADHD.

In the researches of perioperative neurocognitive disorder, scientists testified that an imbalance of excitatory and inhibitory homeostasis mediated by neurotransmitter receptor dysfunction also played an important role in pathogenic mechanisms of cognitive impairments induced by surgery or general anesthesia (19–21). On February 16, 2019, the GAS study clarified that general anesthesia in early infancy did not affect neurodevelopmental outcomes (22). However, whether surgery under general anesthesia was safe for patients with natural neural impairments still remained unclear. Postoperatively, those children with ADHD, comparing with the average person, inclined to exhibit a greater increase in maladaptive behaviors (23). Within another recent clinical research, after strabismus surgery, the relevant symptoms of the children with the ADHD trait were improved (6). Considering these discordances, it was necessary to explore and verify the impacts that surgery and anesthesia exerted on ADHD patients. It was the reason why we focused upon the cognitive performances and ADHD symptoms in this study.

Brain function of ADHD patients were usually deemed as being in the state of overactivation. In our results, the *c-Fos* expression in the NAc area was higher in SHR relative to WKY rats, and this phenomenon could be downregulated by surgery under general anesthesia. Consistent with our results, various changes in the dopaminergic neurotransmission had been shown in SHR (24). Neurons in the NAc area were always categorized as two subtypes, D1R neuron and D2R neuron. A previous study illustrated that neurotransmission mediated by dopamine D1 receptor was elevated in the SHR accumbens (8), which induced overexcitation and hyperactivity symptom. After surgery, the downregulation of neuronal activity in NAc was conducive to reconstructing a new balance in NAc-related circuits through the enhancement of neuroplasticity. For instance, the VTA-NAc-mPFC neuropathway, which was essential for arousal, food intake, memory, locomotor activity, and some other advanced brain functions (25), would be corrected by the de-excitation of NAc *via* surgery under general anesthesia. We would like to proceed with this research by using advanced neural techniques in further studies, such as DREADDs, optogenetic modulation, and so on.

The dynamics of the EEG spectral power was a reflection of brain functional activity. In the current study, we observed that comparing with the preoperation free-moving signal, the power of EEG signal in Delta (0∼3Hz) band became significantly enhanced 1 week after surgery in both SHR and WKY rats, and the band ratio of EEG in delta band was also increased. It suggested that the intrinsic activity pattern of cerebral cortex turned into a new status with much slower wave oscillation after surgery. As we have known, EEG signal power in Delta band used to be generated and enhanced in general anesthesia or NREM sleep (26). In another study, we also found that hyperactivity of ADHD rats subjected to general anesthesia alone could be also improved for at least 1 week, which was paralleled by the variation in Delta band of EEG signal. However, the reasons why EEG change concentrated in Delta band and this phenomenon could last for a long term need further studies.

In conclusion, ADHD individuals were not sensitive to PND. Moreover, surgery with general anesthesia could partly improve the hyperactivity symptom of ADHD. This mechanism was related to the suppression of neural activity in NAc induced by surgery under general anesthesia.

## Data Availability

The raw data supporting the conclusions of this manuscript will be made available by the authors, without undue reservation, to any qualified researcher.

## Ethics Statement

We used the Spontaneously Hypertensive Rats (SHR) as spontaneous ADHD animal model and the Wistar Kyoto Rats (WKY) as non-ADHD animal model. All animal procedures were carried out in accordance with the National Institutes of Health Guide for the Care and Use of Laboratory Animals, and experimental protocols approved by the Ethics Committee for Animal Experimentation of the Fourth Military Medical University. Animals were habituated for 1 week before any intervention with access to water and food *ad libitum*, and kept on a standard 12 h light/12 h dark cycle.

## Author Contributions

PZ contributed to the animal model and behavioral experiments. FX contributed to acquisition and analysis of data and drafting of the manuscript. GZ contributed to animal experiments and drafting of the manuscript. XZ contributed to the EEG experiments. AL contributed to the morphological experiments. HD contributed to the design of experiments and manuscript revision. LX contributed to the conception and design of the study.

## Funding

This work was supported by a key project of the National Science Foundation of China (No. 81730032) to LX, a Support Grant of Xijing Hospital to FX (XJZT18MJ04), and an International Cooperation Grant of Xijing Hospital to GZ (XJZT18RJ09).

## Conflict of Interest Statement

The authors declare that the research was conducted in the absence of any commercial or financial relationships that could be construed as a potential conflict of interest.
